# Nutrient-Driven Adaptive Evolution of Foraging Traits Impacts Producer-Grazer Dynamics

**DOI:** 10.1007/s11538-025-01482-6

**Published:** 2025-06-25

**Authors:** Oluwagbemisola Oladepo, Angela Peace

**Affiliations:** Department of Mathematics and Statistics, Texas Tech University, 2500 Broadway, Lubbock, 79409 TX USA

**Keywords:** Ecological Stoichiometry, Adaptive Dynamics, Foraging strategies

## Abstract

This study investigates the nutrient-driven adaptability of foraging efforts in producer-grazer dynamics. We develop two stoichiometric producer-grazer models: a base model incorporating a fixed energetic cost of feeding and an adaptive model where feeding costs vary over time in response to environmental conditions. By comparing these models, we examine the effects of adaptive foraging strategies on population dynamics. Our adaptive model suggests a potential mechanism for evolutionary rescue, where the population dynamically adjusts to environmental changes, such as fluctuations in food quality, by modifying its feeding strategies. However, when population densities oscillate in predator-prey limit cycles, fast adaptation can lead to very wide amplitude cycles, where populations are in danger of stochastic extinction. Overall, this increases our understanding of the conditions under which nutrient-driven adaptive foraging strategies can yield benefits to grazers.

## Introduction

Foraging plays a critical role in animal fitness, influencing survival, growth, and reproduction (Danchin et al. 2008). Optimal foraging theory employs models to predict animal behaviors that maximize fitness (Pyke et al. 1977). To optimize fitness, animals adopt foraging strategies that provide the most benefit. These strategies are diverse and include compensatory feeding, where animals increase their food intake in response to periods of food restriction or nutritional imbalance. This approach allows grazers to adjust their feeding patterns to meet energetic demands, thereby enhancing survival and fitness. Another important strategy is the adaptability of foraging behaviors over time. As environmental conditions and resource availability change, animals can modify their foraging tactics to maximize energy intake and ensure reproductive success, further improving fitness. These dynamic strategies are essential for optimizing foraging efficiency in fluctuating environments.

This study investigates the nutrient-driven adaptability of foraging traits through a producer-grazer model within the framework of ecological stoichiometry. Ecological stoichiometry explores how the balance of essential elements, such as carbon (C), nitrogen (N), and phosphorus (P), shapes ecological interactions and ecosystem processes (Sterner and Elser 2002). Stoichiometric models integrate the quantity and quality of food resources, examining how nutrient content influences trophic interactions (Loladze et al. 2000; Uszko et al. 2017; Bieg and Vasseur 2024). For grazers, food quality, often represented by the phosphorus-to-carbon ratio (P:C), determines how efficiently they assimilate energy and nutrients. When the P:C ratio is low, grazers may exhibit compensatory foraging, increasing their feeding effort to compensate for nutrient deficiencies (Peace and Wang 2019). When the P:C ratio is high, grazers may adjust foraging rates to limit exposure to excess P levels (Elser et al. 2016; Peace 2015). Notably, Peace and Wang (2019) and Ahmed et al. (2024) have explored compensatory foraging strategies in the context of stoichiometry, where grazers modify their feeding effort in response to nutrient-poor resources. However, the adaptability of foraging behaviors themselves, specifically how grazers adjust their feeding strategies over time, remains underexplored, especially concerning the energetic costs of feeding.

The energetic cost of feeding, the energy expended during foraging activities such as searching, handling, and processing food, varies significantly depending on resource quality. For example, when food is nutrient-poor, grazers may need to expend more energy to process the food efficiently (Elser et al. 2016). This increased feeding cost reduces the energy available for growth, reproduction, and survival, directly impacting fitness. While compensatory foraging strategies have been studied, the adaptability of foraging behaviors themselves–how grazers modify their feeding strategies over time to optimize energy expenditure and nutrient intake in response to changing environmental conditions- remains underexplored.

Modifications in ingestion rates can be modeled as behavioral responses to varying diets. Peace and Wang (2019) assumed foraging efforts directly depended on diet composition and fit foraging functions to data on variable algal nutrient content. On the other hand, empirical studies have investigated the connection between gene expression and the activity of digestive enzymes in *Daphnia*, and transcriptome analyses show a variety of genes coding for gut enzymes are differentially expressed for variable diets (Koussoroplis et al. 2017; Schwarzenberger and Fink 2018). In particular, triacylglycerol lipases and esterase activity, involved in digestion processes, vary with diet composition and can provide a mechanism for physiological adaptations important for foraging efforts (Koussoroplis et al. 2017). Furthermore, it has been shown that digestive traits, such as beta-glucosidase and phosphatase activities are heritable, and coefficients of genetic variation for these digestive traits are higher than those for several life history traits (Tian et al. 2019). Here, we take an evolutionary approach and model foraging efforts adaptively driven by fitness gradients.

Adaptive evolution occurs when natural selection acts on traits within a population, leading to genetic changes that enhance fitness in the current environment. These changes are driven by genetic variations that influence an organism’s ability to survive and reproduce. Traditionally, evolution was viewed as a slow process occurring over long timescales, distinct from the rapid ecological dynamics occurring within ecosystems (Darwin 1859; Slobodkin 1961). However, some studies have shown that evolution can occur on much shorter timescales, influencing ecological processes such as population dynamics, species interactions, and ecosystem functioning (Schoener 2011; Ellner 2013). This process of eco-evolutionary dynamics, which describes the interaction between rapid evolutionary changes and ecological processes, has profound implications for understanding how ecosystems respond to environmental pressures. For example, the evolution of traits such as resource acquisition or predator avoidance can reshape food web interactions and ecological feedback loops (Yamamichi et al. 2015; Yamamichi 2022). As anthropogenic pressures, such as climate change and habitat destruction, intensify, the role of eco-evolutionary dynamics becomes increasingly significant in shaping ecosystem responses (Matthews et al. 2011).

Eco-evolutionary dynamics are closely linked to the field of ecological stoichiometry. Ecological stoichiometry is crucial for understanding how nutrient availability and limitations impact foraging behavior, growth, and reproduction. Nutrient limitations–such as low phosphorus or nitrogen availability–force organisms to adjust their physiological, behavioral, or evolutionary strategies to maintain fitness. Therefore, understanding how foraging behavior, like a grazer’s feeding effort, evolves in response to nutrient limitations is key to understanding broader ecological processes.

To examine the effects of feeding efforts, we develop a producer-grazer model within the framework of ecological stoichiometry (Sterner and Elser 2002). We first develop a base model by modifying the functional form of the grazer’s ingestion rate and incorporating a fixed energetic cost for feeding efforts into the stoichiometric producer-grazer model introduced by Loladze et al. (2000). This modification accounts for baseline energy expenditures associated with foraging and allows us to explore how feeding efforts affect population dynamics. We then introduce an adaptive component to the model, allowing the energetic cost of feeding to evolve over time in response to a fitness gradient. Using methods from Adaptive Dynamics (Cortez and Ellner 2010), we examine how grazers adjust their foraging expenditure under fluctuating environmental conditions. Analyzing and comparing these two models allows us to investigate the potential benefits of foraging effort adaptation for the grazer population.

## Model Development

In this section, we formulate models of the first two trophic levels of an aquatic food chain, consisting of primary producer *x* (algae, mg C/L) and grazer *y* (Daphnia, mg C/L) biomass density. We assume that both the producer and the grazer contain essential elements- carbon (C) and phosphorus (P)- and that their growth depends on these nutrients.

The models assume that the grazer has a Holling type II functional response and directly incorporates the carbon cost of feeding effort. First, we present a base model (Section 2.1) where the grazer’s carbon cost of feeding is fixed, and then we develop an adaptive model (Section 2.2) where the grazer’s carbon cost of feeding varies over time.

### Base Model

The base model takes the following form: 1a1b Here, b is the maximum growth rate of the producer, K is the producer carrying capacity in terms of light, P is the total phosphorus in the system, q is the producer minimum P:C ratio required for survival, $\theta$ is the grazer minimum P:C ratio needed for survival, $\hat{e}$ is the grazer maximum conversion efficiency, and d is the grazer loss rate. Additionally, the feeding effort $\xi$ is taken as a fixed cost of biomass, representing the energetic expenditure associated with foraging activity. In particular, higher feeding efforts can lead to increased respiration costs due to greater mobility and the physiological demands of food processing. This trade-off captures the balance between energy gain and metabolic loss, which is crucial in ecological stoichiometry. Similar formulations have been used in studies examining optimal foraging under stoichiometric constraints (Suzuki-Ohno et al. 2012), where compensatory feeding strategies result in increased energy expenditure that may offset the benefits of higher ingestion rates.

Similar to previous models developed under the framework of ecological stoichiometry (Loladze et al. 2000), our models employ a nonsmooth minimum function in the growth rate expressions, which reflects Liebig’s law of the minimum. According to this law, an organism’s growth will be limited by whichever single resource is in the lowest supply relative to the organism’s needs (Sterner and Elser 2002). In this context, we assume the growth rates are limited by either C or P. This results in the minimum functions with two inputs seen in the growth rate expressions in model (1).

The model assumes that the grazer has a Holling type II functional response, f(x), and directly takes into account the carbon cost of feeding effort. Following Suzuki-Ohno et al. (2012), we assume that the encounter rate with the producer increases linearly with feeding effort, and *f*(*x*) takes the form:

2 $$\begin{aligned} f(x)= \frac{\mu \xi x}{1+\mu \xi \tau x} \end{aligned}$$

where $\mu$ is the amount of water cleared per mg C invested to generate energy for filtering behavior and $\tau$ is the handling time. This base model (1) is similar to the LKE model developed by Loladze et al. (2000) with the addition of the grazer’s cost of feeding, $\xi$, and a modified functional response *f*(*x*) given in equation (2). This model assume the system is closed with respect to P, and all the P is in the producer or in the grazer. Additionally it assumes that the producer has a variable P:C ratio given by the expression $\frac{P-\theta y}{x}$ and the grazer has a constant P:C ratio $\theta$.

### Adaptive Model

In this model, we build upon the quantitative genetic approach to explore population dynamics (Fussmann et al. 2007; Cortez and Ellner 2010). This approximation from the fitness gradient follows from quantitative genetics. We assume that the grazer’s feeding effort is a stoichiometric trait that undergoes rapid evolution in response to producer stoichiometry and changes at a rate proportional to the additive variance of the trait and the fitness gradient.

3 $$\begin{aligned} \frac{d\xi }{dt}= (\xi _{max}-\xi )(\xi - \xi _{min})v\frac{\partial W}{\partial \xi } \end{aligned}$$

where *W* is the per capita growth rate (a proxy for fitness) of the grazer, $W= \frac{1}{y}\frac{dy}{dt}$, *v* is the additive genetic variance of $\xi$. The partial derivative of *W* is the selection gradient and therefore $\xi$ changes adaptively to increase *W*. The speed of adaptation is determined by *v*, which can be parameterized for models of rapid evolution so that adaptive evolution occurs on the same timescale as population dynamics (Hairston et al. 2005). Parameters $\xi _{max}$ and $\xi _{min}$ are the maximum and minimum grazer’s feeding cost. Following Cortez and Ellner (2010), the expression $(\xi _{max}-\xi )(\xi - \xi _{min})$ was used in order to ensure that $\xi$ remains within biologically realistic range.

The system of equations governing this adaptive model is given in model (4). The equations describe the time evolution of the producer, *x*(*t*), and grazer, *y*(*t*), population densities, and the feeding effort trait $\xi (t)$. The adaptive model takes the form: 4a4b4c

## Model Analysis

In this section, we present an analysis of both models, verifying the boundedness and invariance of their solutions. We locate boundary equilibria, develop criteria to determine their stability, and numerically investigate dynamics near interior equilibria to determine their local stability.

### Base Model

#### Boundedness and Invariance

For this model, the carrying capacity of x depends on the density of y. In the absence of the grazer y, the carrying capacity of producer x depends only on light and phosphorus availability, which we denote as

5 $$\begin{aligned} k = \min \left( K, \frac{P}{q}\right) \end{aligned}$$

The model is well defined as $x\rightarrow {0}$: *f*(*x*) is a bounded smooth function that satisfies the following assumptions:

6 $$\begin{aligned} f(0) = 0, f'(x)>0, f'(0)<\infty \,\, and\,\, f''(x)<0 \,\, \forall \, x\ge 0 \end{aligned}$$

Making these assumptions ensures that $\frac{f(x)}{x}$ has the following properties

7 $$\begin{aligned} \lim _{x \rightarrow {0}} \frac{f(x)}{x} = f'(0)< \infty \text { and } \left( \frac{f(x)}{x}\right) '<0 \quad \forall x>0 \end{aligned}$$

As $x\rightarrow {0}$, $\frac{dy}{dt}$ is well defined and this is because

$$\min \left( 1,\frac{(P-\theta y)/x}{\theta }\right) f(x) = \min \left( f(x),\frac{(P-\theta y)}{\theta }\frac{f(x)}{x}\right) $$

If the total amount of Phosphorus is bounded in the system, and both populations require it, then their densities should be bounded as well.

##### Theorem 1

Solutions with initial conditions in the open rectangle

8 $$\begin{aligned} \Delta = \{(x,y):0<x<k=\min \left( K, \frac{P}{q}\right) ,\quad 0<y<\frac{P}{\theta }\} \end{aligned}$$

will remain there for all forward time.

##### Proof

Let (*x*(*t*), *y*(*t*)) be a solution of system (1) with initial conditions $(x(0),y(0) \in \Delta$. Assume, for contradiction, that there exists a time $t_1>0$, such that the trajectory with initial conditions in the open rectangle touches or crosses the boundary of the closed rectangle for the first time.

The boundary of the closed rectangle consists of four sides, so four cases are possible:

Case 1: $x(t_1) = 0$: We assume that the trajectory reaches the left side of the rectangle.

Let $y_1 = \max _{t\in [0,t_1]} y(t)<\frac{P}{\theta }$ and $\hat{f}=f'(0)=\lim _{x\rightarrow {0}}\frac{f(x)}{x}$ . Then for every $t\in [0,t_1]$,

$$\begin{aligned} x'&= bx\min \left( 1-\frac{x}{K}, 1-\frac{x}{(P-\theta y)/q}\right) - f(x) y \\&\ge bx\min \left( 1-\frac{x}{K}, 1-\frac{x}{(P-\theta y)/q}\right) - \hat{f}xy\\&\ge \left( b\min \left( 1-\frac{k}{K}, 1-\frac{k}{(P-\theta y_1)/q}\right) - \hat{f}y_1\right) x \equiv \delta x\\ \end{aligned}$$

where $\delta$ is a constant.

The differential equation simplifies to:

$$x(t)\ge x(0)e^{\delta t}$$

This implies that $x(t_1)\ge x(0)e^{\delta t_1}>0$, contradicting the assumption that $x(t_1)=0$. Thus, no trajectory can touch the left-hand-side boundary of the rectangle.

The proof proceeds similarly for the other three cases, where the trajectory touches the remaining sides of the rectangle, showing that no trajectory can touch any boundary, and is presented in Appendix A.1. $\square$

#### Equilibria of the Base Model

For the boundary equilibria, we consider the system,

9a $$\begin{aligned} \frac{dx}{dt}&= xF(x,y) \end{aligned}$$

9b $$\begin{aligned} \frac{dy}{dt}&= yG(x,y) \end{aligned}$$

where

$$\begin{aligned} F(x,y)&= b\min \left( 1-\frac{x}{K}, 1-\frac{x}{(P-\theta y)/q}\right) - \frac{f(x)}{x} y \\ G(x,y)&= \hat{e}\min \left( 1,\frac{(P-\theta y)/x}{\theta }\right) f(x) - \xi - d \end{aligned}$$

and

$$f(x)=\frac{\mu \xi x}{1+\mu \xi \tau x}$$

There are two equilibria on the boundary: $E_0 = (0,0)$, where both the producer and grazer face extinction, and $E_1 = (k,0)$, where only the grazer does not persist. The following theorems provide results on the stability of these equilibria.

The Jacobian of the above system (9) is given by

$$ \textbf{J} = \begin{bmatrix} F(x,y) + xF_x(x,y) & xF_y(x,y) \\ yG_x(x,y) & G(x,y) + yG_y(x,y) \end{bmatrix} $$

##### Theorem 2

The boundary extinction equilibrium $E_0=(0,0)$ is unstable.

##### Proof

The Jacobian matrix at $E_0$,

$$ \mathbf {J|_{E_0}} = \begin{bmatrix} F(0,0) & 0 \\ 0 & G(0,0) \end{bmatrix} $$

$$ = \begin{bmatrix} b & 0 \\ 0 & -\xi -d \end{bmatrix} $$

The eigenvalues are *b* and $-(\xi +d)$.

Therefore, $E_0$ is a saddle point and it is always unstable. $\square$

##### Theorem 3

The boundary grazer extinction equilibrium $E_1=(k, 0)$ is locally asymptotically stable if

$$G(k,0)=\hat{e}\min \left( 1,\frac{P}{k\theta }\right) \left( \frac{\mu \xi k}{1+\mu \xi \tau k}\right) - \xi - d<0.$$

where $$k=\min \left( K,\frac{P}{q} \right) $$

##### Proof

The Jacobian matrix at $E_1$,

$$ \mathbf {J|_{E_1}} = \begin{bmatrix} F(k,0) + kF_x(k,0) & kF_y(k,0) \\ 0 & G(k,0) \end{bmatrix} $$

$$ = \begin{bmatrix} -b & kF_y(k,0) \\ 0 & G(k,0) \end{bmatrix} $$

where

10 $$\begin{aligned} F_y(k,0) = {\left\{ \begin{array}{ll} \displaystyle -\frac{\mu \xi }{1+\mu \xi \tau k}\  & \text {if } K<\frac{P}{q} \\ \\ \displaystyle -\frac{bkq\theta }{P^2}-\frac{\mu \xi }{1+\mu \xi \tau k} & \text {if }K>\frac{P}{q} \end{array}\right. } \end{aligned}$$

and

11 $$\begin{aligned} G(k,0) = \hat{e}\min \left( 1,\frac{P}{k\theta }\right) \left( \frac{\mu \xi k}{1+\mu \xi \tau k}\right) - \xi - d \end{aligned}$$

The local stability of $E_1$ is determined by the sign of *G*(*k*, 0). If *G*(*k*, 0) is positive, $E_1$ is unstable, and if *G*(*k*, 0) is negative, $E_1$ is locally asymptotically stable. $\square$

The condition $G(k,0)<0$ in Theorem 3 implies that the energetic cost of feeding and the natural death rate combined are greater than the grazer’s reproductive growth rate at the equilibrium $E_1$, where the grazer population is extinct. Here, the grazer dies out and equilibrium $E_1$ is stable because its population will decline under these conditions and cannot avoid extinction. Regions across light level and feeding effort parameter space where the grazer dies out and $E_1$ is stable are showcased in Appendix A.2, where we plotted *G*(*k*, 0) for varying light level (*K*) and feeding effort ($\xi$).

For interior equilibria, where both species persist we explore phase plane analysis for varying values of *K*. These showcase the existence and stability of positive interior equilibria (see appendix A.3).

### Adaptive Model

#### Theorem 4

Solutions with initial conditions in the open set

12 $$\begin{aligned} \Delta = \{(x,y,\xi ):0<x<k=\min \left( K, \frac{P}{q}\right) ,\quad 0<y<\frac{P}{\theta },\quad \xi _{min}\le \xi \le \xi _{max}\} \end{aligned}$$

will remain there for all forward time.

See proof of this theorem in Appendix B.1

#### Equilibria of the Adaptive Model

For the boundary equilibria, we rewrite the adaptive model (4) in the following form:

13a $$\begin{aligned} \frac{dx}{dt}&= xF(x,y,\xi ) \end{aligned}$$

13b $$\begin{aligned} \frac{dy}{dt}&= yG(x,y,\xi ) \end{aligned}$$

13c $$\begin{aligned} \frac{d\xi }{dt}&= vH(x,y,\xi ) \end{aligned}$$

where

$$\begin{aligned} F(x,y,\xi )&= b\min \left( 1-\frac{x}{K}, 1-\frac{x}{(P-\theta y)/q}\right) - \left( \frac{\mu \xi }{1+\mu \xi \tau x}\right) y \\ G(x,y,\xi )&= \hat{e}\min \left( 1,\frac{(P-\theta y)/x}{\theta }\right) \left( \frac{\mu \xi x}{1+\mu \xi \tau x}\right) - \xi - d \\ H(x,y,\xi )&= (\xi _{max}-\xi )(\xi - \xi _{min})\left[ \hat{e}\min \left( 1,\frac{(P-\theta y)/x}{\theta }\right) \frac{\mu x}{(1+\mu \xi \tau x)^2} - 1 \right] \end{aligned}$$

There are equilibria on the boundary: $E_0 = (0,0,\xi ^*)$, where both the producer and grazer face extinction, and $E_1 = (k,0,\xi ^*)$, where only the grazer does not persist.

The following theorems provide results on the stability of these equilibria. The Jacobian of the above system (13) is given by

$$ \textbf{J} = \begin{bmatrix} F(x,y,\xi ) + xF_x(x,y,\xi ) & xF_y(x,y,\xi ) & xF_{\xi }(x,y,\xi ) \\ yG_x(x,y,\xi ) & G(x,y,\xi ) + yG_y(x,y,\xi ) & yG_{\xi }(x,y,\xi ) \\ vH_x(x,y,\xi ) & vH_y(x,y,\xi ) & vH_{\xi }(x,y,\xi ) \end{bmatrix} $$

##### Theorem 5

The boundary equilibria where both the producer and grazer face extinction, $E_0= (0,0,\xi ^*)$, is unstable.

##### Proof

At $E_0$, the Jacobian matrix takes the form

$$ \mathbf {J|_{E_0}} = \begin{bmatrix} b & 0 & 0 \\ 0& -d-\xi ^* & 0 \\ 0 & 0 & (\xi ^*-\xi _{min})v-(\xi _{max}-\xi ^*)v \end{bmatrix} $$

The eigenvalues of $\mathbf {J|_{E_0}}$ have different signs. Thus, $\mathbf {{E_0} = (0,0,\xi ^*)}$ is always unstable.

$$\square $$

##### Theorem 6

A grazer extinction equilibrium of the form $E_1= (k,0,\xi ^*)$ is stable if

$$\begin{aligned} G(k,0,\xi ^*)&= \hat{e}\min \left( 1,\frac{P}{k\theta }\right) \left( \frac{\mu \xi ^* k}{1+\mu \xi ^*\tau k}\right) - \xi ^* - d<0 \text { and, }\\ H_{\xi }(k,0,\xi ^*)&= -(\xi ^*-\xi _{min})\left[ \frac{\hat{e}\min \left( 1,\frac{P}{k\theta }\right) \mu \xi ^*}{\left( 1+\mu \xi ^*\tau k\right) ^2}-1\right] + (\xi _{max}-\xi ^*)\left[ \frac{\hat{e}\min \left( 1,\frac{P}{k\theta }\right) \mu \xi ^*}{\left( 1+\mu \xi ^*\tau k\right) ^2}-1\right] \\&+ (\xi _{max}-\xi ^*)(\xi ^*-\xi _{min})\left[ \hat{e}\min \left( 1,\frac{P}{k\theta }\right) \right] \left[ \frac{\mu -\mu ^2\xi ^*\tau k}{(1+\mu \xi ^*\tau k)^3}\right] <0. \end{aligned}$$

where $$k=\min \left( K,\frac{P}{q}\right) $$.

The proof of this theorem can be found in Appendix B.2

The conditions $G(k, 0, \xi ^*) < 0$ and $H_{\xi }(k, 0, \xi ^*) < 0$ in Theorem 6 imply that the grazer extinction equilibrium is stable. Biologically, the condition $G(k, 0, \xi ^*) < 0$ indicates that the net growth rate of the grazer is negative at equilibrium: the grazers cannot persist because the energy or nutrient gain from foraging is insufficient to compensate for losses from mortality and the energetic cost of feeding. The second condition, $H_{\xi }(k, 0, \xi ^*) < 0$, is helpful in determining whether the grazer extinction equilibrium $E_1 = (k, 0, \xi ^*)$ is stable. Appendix B.3 shows visualizations of this condition for varying light level, *K*, and $\xi ^*$.

For interior equilibria of the adaptive model, where both species persist, we numerically investigate the stability of the equilibria with various initial conditions (see Appendix B.4).

## Numerical Experiments

This section presents the findings from numerical simulations and a numerical bifurcation analysis on interior equilibria. The parameter values used for the model are listed in Table 1.

**Table 1:** Most parameters are values obtained from Peace and Wang (2019); Suzuki-Ohno et al. (2012) except for parameters $\xi$ and *v* which we varied across the ranges shown

Parameter		Value	Units
P	Total phosphorus	0.025	mg P/l
$\hat{e}$	Maximal C production efficiency	0.8	
b	Producer maximal growth rate	1.2	$\hbox {day}^{-1}$
d	Grazer loss rate	0.2	$\hbox {day}^{-1}$
$\theta$	Grazer constant P:C	0.03	(mg P)/(mg C)
q	Producer minimal P:C	0.0038	(mg P)/(mg C)
K	Producer light-dependent carrying capacity	0.25 - 2	mg C/l
$\mu$	Foraging water cleared/mg C	700	l/mg C
$\tau$	Handling time	1.23	$\hbox {day}^{-1}$
$\xi$	Feeding effort, constant for base model	0.004 - 0.01	mgC/mgC/d
*v*	Speed of adaptation	0.001 - 0.1	

### Numerical Simulations

**Fig. 1:**
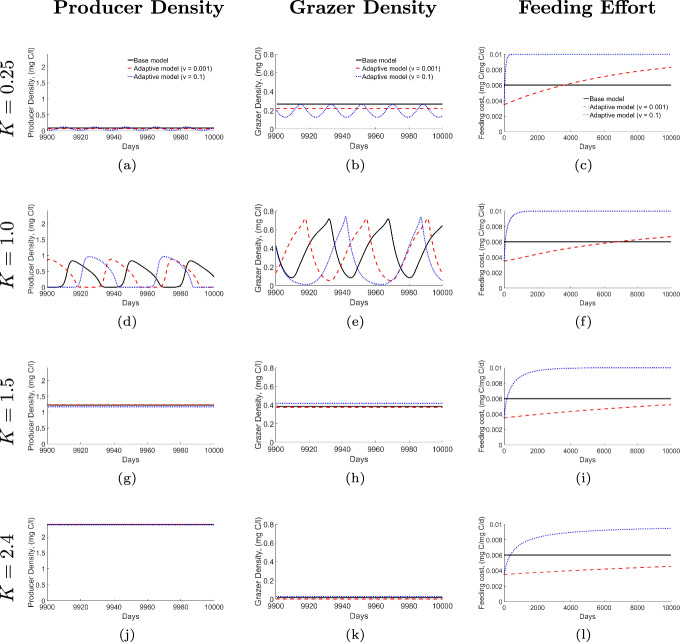
Numerical simulations for the base model (solid black line) and the adaptive model with low adaptation speed (red dashed line) and high adaptation speed (blue dotted line) under different light intensities. Each row shows Producer Density, Grazer Density, and Feeding Effort for specific values of $K$: (a)(b)(c) $K=0.25$ mgC/l, (d)(e)(f) $K=1.0$ mgC/l, (g)(h)(i) $K=1.5$ mgC/l, and (j)(k)(l) $K=2.4$ mgC/l

Figure 1 presents numerical simulations of the base model (1) and adaptive model (4) with low speed of adaptation ($v=0.001$) and high speed of adaptation ($v=0.1$). Simulations were conducted for 10,000 days, with the last 100 days plotted to showcase the long-term dynamics. Each row represents different light intensities, with the corresponding values of *K* indicated on the left (from $K=0.25$ mgC/l to $K=2.4$ mgC/l)

In Fig. 1, the population density dynamics, displayed in the first and middle columns, vary with increasing light intensity ($K$). At $K = 0.25$ mg C/l, population densities remain stable over time in both the base model (black line) and the adaptive model with low adaptation speed (red dashed line), while the adaptive model with high adaptation speed (blue dotted line) shows oscillatory behavior (Fig. 1ab). When the light intensity increases to $K = 1.0$ mg C/l, the population densities show oscillations in both models. Both models show cyclic fluctuations, but the oscillations are more pronounced in the adaptive model with high adaptation speed (blue dotted line), which has a larger amplitude than the others (Fig. 1de). At $K = 1.5$ mg C/l, oscillations cease, and population densities stabilize in all models. At the higher rate of adaptation, the adaptive model shows a higher grazer density compared to both the base model and the adaptive model with slower adaptation (Fig. 1gh). Finally, at high light levels of $K = 2.4$ mg C/l, the population densities remain stable, similar to the behavior observed at $K = 1.5$ mg C/l. However, despite the abundance of food, grazer densities in all models approach extinction. This occurs because the quality of food decreases with increasing $K$, making it unsustainable for the grazer population (Fig. 1jk).

As we vary the light intensity in the population dynamics, we also see changes in the feeding efforts displayed in the last column of Fig. 1. Generally, feeding effort increases over time for the adaptive model. At high speeds of adaptation ($v=0.1$ blue curve) the grazer is quicker to invest effort into feeding than at lower speeds of adaptation ($v=0.001$ red curve) across all cases. As light increases, the rate at which the feeding effort $\xi$ increases slows down (Fig. 1 third column ).

Although the feeding effort is high for the adaptive model (with $v = 0.1$), the grazer’s biomass remains low in Fig. 1(b), where light levels are low. This occurs because the available food (the producer biomass) is also low in this scenario, as shown in Fig. 1(a). However, when food availability increases with increasing light levels, higher feeding effort can be beneficial to the grazers, as seen in Fig. 1(h), where higher feeding efforts lead to a higher grazer biomass under better food conditions. On the other hand, when light levels reach very high values, the grazer’s biomass remains low (fig. 1(k)) despite high feeding efforts (Fig. 1(l)). This occurs because the available food, while high in quantity (fig. 1(j)), is low in quality, and the grazers are nutrient-limited. Thus, both low food quantity and low food quality can limit grazer success, even when the foraging effort is high.

### Bifurcation Analysis

We conducted a numerical bifurcation analysis to investigate the long-term behavior of the grazer population in the base model as the producer’s carrying capacity $K$ changes across different values of the feeding effort $\xi$. This analysis provides insight into how variations in $K$ influence the grazer population over time and how an increase in feeding cost affects the dynamics of the grazer population. Additionally, we performed a bifurcation analysis on the adaptive model to explore the long-term behavior of both the grazer population and their feeding cost as $K$ varies, considering different adaptation speeds. Each model was run for $10{,}000$ days.

**Fig. 2:**
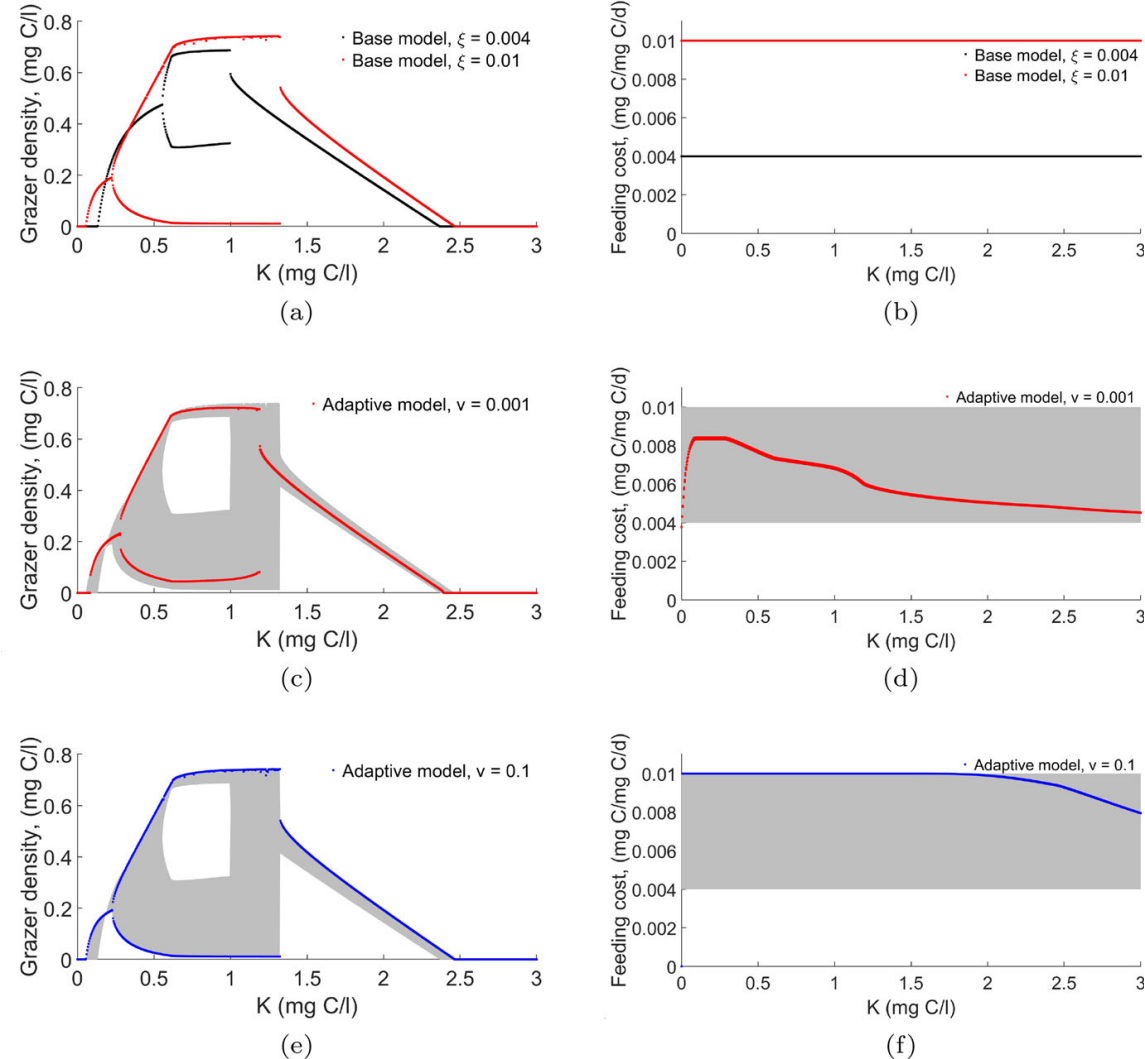
Bifurcation diagrams illustrating the dynamics of the base and adaptive models under varying light-dependent carrying capacity, $K$. (a) shows the grazer population as $K$ varies for different feeding effort ($\xi$) values in the base model, while (b) shows the corresponding feeding efforts. (c) and (d) depict the grazer population for the adaptive model under low ($v = 0.001$) and high ($v = 0.1$) speeds of adaptation, respectively. The shaded region in (c), (d), (e), and (f) highlights the area between the bifurcation diagrams for the base model with low feeding cost ($\xi = 0.004$ mgC/mgC/d) and high feeding cost ($\xi = 0.01$ mgC/mgC/d) in (a) and (b)

As observed in Fig. 2(a), at a low feeding cost ($\xi = 0.004$ mgC/mgC/d), our base model shows that the grazer population cannot survive at low $K$ values due to insufficient food availability. As $K$ increases, the grazer population begins to grow. This growth is initially steady, but when a Hopf bifurcation occurs, the population enters a limit cycle, resulting in oscillations between high and low population densities. As $K$ increases further, these cycles stop, and the population stabilizes. However, at the point where the cycles end, the population encounters a saddle-node bifurcation where a new equilibrium gains stability. As *K* continues to increase, the grazer density declines and ultimately goes to extinction. This decline occurs despite an abundance of food, as the quality of food decreases with increasing $K$, making it unsustainable for the grazer population.

When the feeding effort increases to $\xi = 0.01$ mgC/mgC/d, as shown in Fig. 2(a), the bifurcation diagram retains a similar structure to the low feeding cost case, but the limit cycles emerge sooner and are larger. However, higher feeding efforts allow the grazer population to persist in conditions where it would otherwise face extinction. This comes with a trade-off; while higher feeding efforts result in greater fluctuations (larger limit cycles), they also confer a survival advantage, enabling persistence in conditions that would otherwise lead to extinction. In this scenario, increased feeding efforts act as a form of evolutionary rescue, enabling the grazer population to survive in conditions that would otherwise lead to extinction.

Next, we explore the bifurcation diagram of our adaptive model, where the grazer population’s feeding cost can vary over time. In Fig. 2 (c, d, e, and f), the shaded region highlights the area between the bifurcation diagrams for the base model with low feeding cost ($\xi = 0.004$ mgC/mgC/d) and high feeding cost ($\xi = 0.01$ mgC/mgC/d) in Fig. 2(a and b). We shade the region to compare with the adaptive model at low ($v = 0.001$) and high ($v = 0.1$) speeds of adaptation.

At a low adaptation speed ($v = 0.001$), the grazer density bifurcation diagram (Fig. 2c) follows a similar structure as the base model. However, the feeding cost now varies adaptively over time (Fig. 2d). This adaptive variation in feeding cost enables the grazer population to dynamically respond to changes in $K$, allowing it to persist under conditions that would otherwise lead to extinction in the base model. The shaded region in Fig. 2(d) represents the range of feeding efforts observed in the base model at low and high feeding efforts, indicating the flexible range within which the adaptive model operates. Comparing the bifurcation diagram of grazer density at a low adaptation speed with the shaded region highlights how adaptability reduces the risk of stochastic extinction. This effect is particularly evident in small regions at low values of $K$ ($0.081< K < 0.14$), where resource scarcity is a critical challenge, and at high values of $K$ ($2.35< K < 2.4$), where poor food quality might otherwise drive the population to extinction.

In Fig. 2(e and f), we examine the effects of a higher adaptation speed ($v = 0.1$). In this case, the grazer density bifurcation diagram (Fig. 2e) still follows a similar pattern, but the amplitude of the limit cycles increases, reflecting a more rapid response of the population to changes in $K$. Fig. 2(f) shows that the feeding cost adapts more quickly than in the low-speed case, allowing the population to adjust to environmental changes more effectively. The shaded regions in Fig. 2(d and f) illustrate the variability in feeding efforts for the adaptive model compared to the base model, demonstrating how the grazer population can dynamically adjust feeding efforts within the range defined by both low and high feeding efforts in the base model. The faster adaptation rate causes the grazer population to react more quickly to environmental fluctuations, which could increase the likelihood of stochastic extinction.

On the other hand, in comparison to slow adaptation (Fig. 2 c and d), faster adaptation (Fig. 2 e and f) may offer benefits. It helps sustain grazer density more effectively at both low and high values of $K$, enabling the population to maintain stability in environments that could otherwise lead to extinction. The faster adaptation rate improves the population’s ability to adjust its feeding strategies quickly, which supports survival under changing conditions. In this context, the adaptive feeding cost serves as a form of evolutionary rescue. By modifying its feeding behavior, the population increases its chances of survival in fluctuating environments, helping to reduce the risks that could lead to random extinction. While the base model assumes fixed feeding efforts, the adaptive model shows that allowing the feeding cost to vary over time can enable the population to survive in conditions that would otherwise be detrimental, highlighting the potential for adaptive traits to act as a form of evolutionary rescue.

**Fig. 3:**
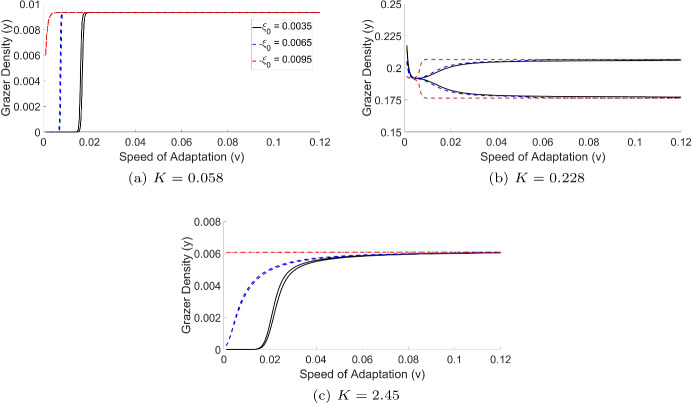
Long term dynamics showing grazer biomass after $t=10,000$ days under varying speed of adaptation for three fixed values of producers light-dependent carrying capacity *K*: (a) low $K=0.058$ mg C/l, (b) intermediate $K=0.228$ mg C/l, and (c) high $K=2.45$ mg C/l. For each subfigure, three initial ($t=0$) feeding efforts $\xi _0 = 0.0035$ (black), $\xi _0 = 0.0065$ (blue), and $\xi _0 = 0.0095$ (red) are shown. For each $\xi _0$, the curves represent the minimum and maximum grazer densities over the final 500 days of simulation, capturing the range of long-term dynamics under different adaptation speeds and initial trait conditions

To further explore how the speed of adaptation affects grazer density, we performed a one-parameter bifurcation analysis of the adaptive model by varying the speed of adaptation $v$ at fixed values of light level $K$ and across different initial values of the feeding effort trait $\xi _0$. The results (Fig. 3) show that grazer biomass responds differently to increases in $v$, depending on the environmental conditions. At low $K$ (Fig. 3a), grazers starting off with low $\xi _0$ cannot persist for small $v$, but once a critical threshold is crossed, biomass increases sharply, indicating that rapid adaptation is necessary to survive under strong food limitations. This transition is most dramatic for the black curve ($\xi _0 = 0.0035$), where the trait begins far from the optimal value, and less so for the red curve ($\xi _0 = 0.0095$), which starts closer to optimal feeding efficiency. At high $K$ (Fig. 3c), biomass increases more gradually with $v$, reflecting the grazer’s ability to adjust to poor food quality. In this case, the blue and red curve shows that when the grazer starts closer to its optimal feeding effort, high biomass can be maintained even at low adaptation speeds. This suggests that rapid adaptation is less essential when the initial trait is already well-suited to the environment. At an intermediate value of $K$ (Fig. 3b), changes in $v$ can shift populations from equilibria to oscillatory dynamics.

## Conclusion

This study examines two models of producer-grazer dynamics. The base model is a stoichiometric model that incorporates a Holling type II functional response and the grazer’s carbon cost of feeding efforts, as proposed by Suzuki-Ohno et al. (2012). Our findings show that increasing the feeding cost not only constrains population growth but also introduces a trade-off. This trade-off enables grazers to persist under conditions that would otherwise lead to extinction, particularly when resources are abundant but of lower quality. Thus, higher feeding efforts act as a mechanism of evolutionary rescue, promoting survival in challenging environments despite introducing larger population fluctuations. However, the trade-off occurs when population densities oscillate in predator-prey limit cycles, and adaptation can lead to very wide amplitude cycles, where populations are in danger of stochastic extinction.

Recognizing that fixed feeding efforts may not fully capture ecological reality, we extended the base model to include an adaptive feeding cost in the adaptive model. Here, the feeding effort evolves adaptively over time in response to population and environmental changes. Comparing the two models reveals that the adaptive model can provide significant benefits to grazers, particularly in dynamic environments. The flexibility of varying feeding efforts allows grazers to better withstand resource scarcity or poor food quality, reducing extinction risks in certain regions. However, adapting too fast can have larger amplitude cycles where the grazers are in danger of stochastic extinction.

This study primarily addresses compensatory foraging, where grazers adjust the amount of food they consume to meet energetic needs. While this mechanism is central to both models, it does not explore complementary foraging, where grazers alter the composition of their diet by switching between resource types. Incorporating complementary foraging could provide further insight into how grazers adapt to environments with varying resource quality and diversity. Additionally, while we considered a Holling type II functional response throughout our base and adaptive models, it has been shown that using Holling type III functional responses in stoichiometric producer-grazer models can affect population dynamics (Uszko et al. 2017).

The interactions of varying temperature and nutrient stressors can impact stoichiometric constraints (Bieg and Vasseur 2024; Uszko et al. 2017). Extending our adaptive model to environments with varying temperatures may shift bifurcation dynamics and regions where trajectories approach equilibria, as shown in Bieg and Vasseur (2024). Thermal response curves can affect growth rates, and including temperature-dependent foraging behaviors processes may also be insightful.

Overall, this study highlights the importance of adaptiveness in feeding strategies. The base model illustrates how fixed costs shape population dynamics, while the adaptive model demonstrates the evolutionary advantage of dynamic feeding strategies and the potential consequences of adapting too quickly. This insight provides a deeper understanding of the conditions under which adaptive behaviors benefit grazers, offering a foundation for future exploration of ecological resilience and evolutionary adaptation.

## Figures and Tables

**Fig. 4:**
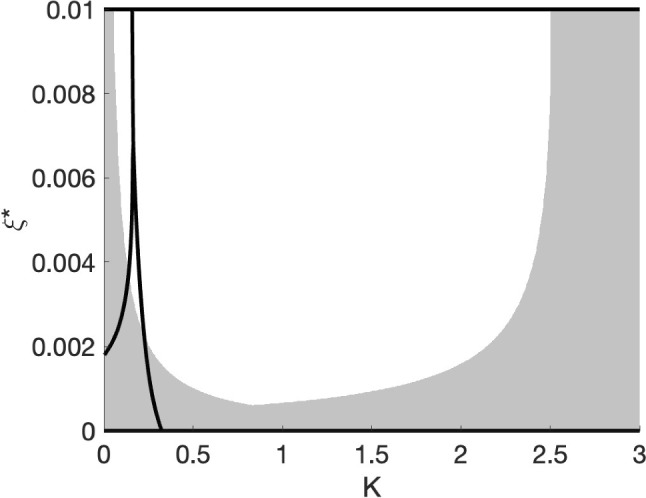
Illustration of the region in $(K, \xi ^*)$ parameter space where $G(k, 0) < 0$ is satisfied. The light gray region indicates areas where the condition holds, ensuring the possible stability of the grazer extinction equilibrium $E_1$, while the white region represents areas where the condition is not satisfied, indicating $E_1$ is unstable. Possible equilibrium points $E_1$ will lie on the black curve where $\frac{d\xi }{dt} = 0$

**Fig. 5:**
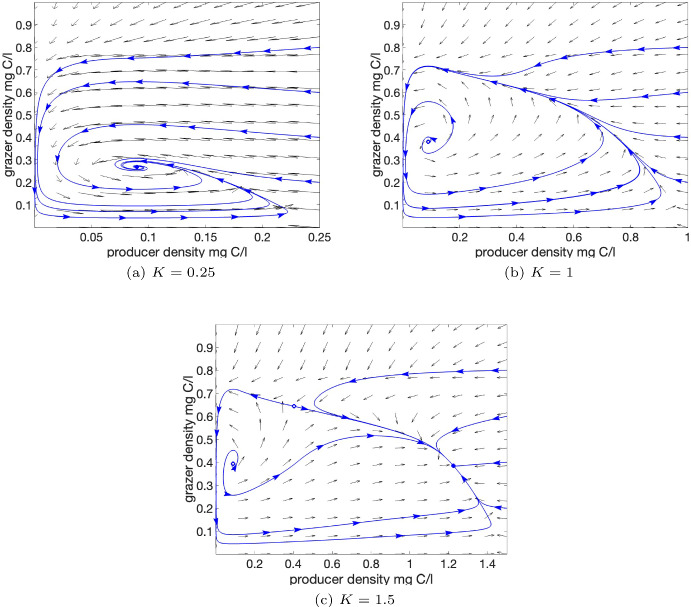
Phase plane for the base model 1 for different light intensities. Sample trajectories are shown in blue curves with arrows in forward time. Interior equilibria are shown as open (unstable) and filled (stable) blue circles

**Fig. 6:**
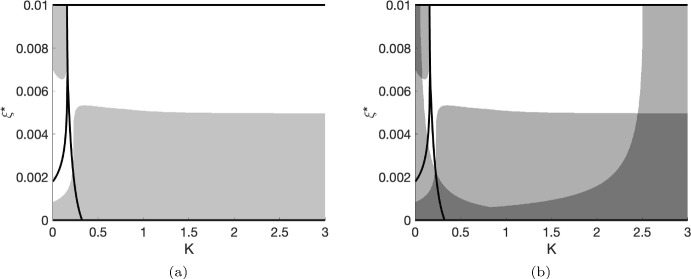
Illustrations of the region in $(K, \xi ^*)$ parameter space where $H_{\xi }(k, 0, \xi ^*) < 0$ is satisfied (a) and area where both conditions: $G(k, 0, \xi ^*) < 0$ and $H_{\xi }(k, 0, \xi ^*) < 0$ are satisfied (b). The light gray regions in a and b indicates areas where the individual conditions hold, the white region represents areas where neither condition is satisfied, and the dark gray region indicates where both conditions hold, ensuring the stability of the grazer extinction equilibrium. Possible equilibrium points $E_1$ will lie on the black curve where $\frac{d\xi }{dt} = 0$

**Fig. 7:**
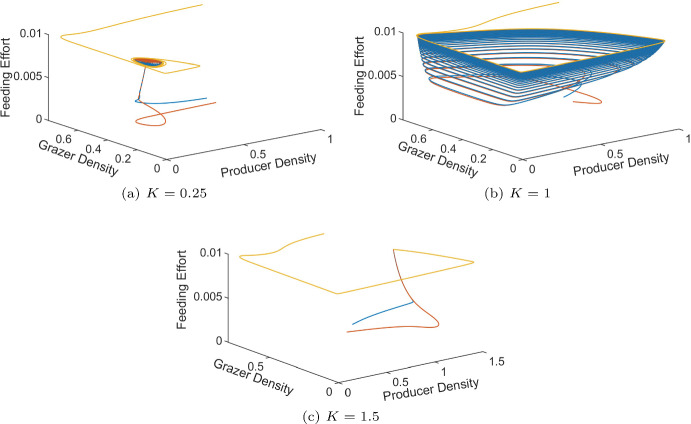
Phase portrait for adaptive model 4 for different light intensities $K$. (a) $K = 0.25$, (b) $K = 1$, and (c) $K = 1.5$ show the phase portraits of the producer and grazer densities along with the corresponding feeding effort. The trajectories are plotted for different initial conditions, illustrating how the system evolves under different environmental conditions and light levels

## Base Model Analysis

### Remaining Cases for Proof of Theorem 1

#### Proof

Case 2: $x(t_1) = k=\min \left( K,\frac{P}{q}\right)$. We assume that the trajectory reaches the right side of the rectangle. Then for every $t\in [0,t_1]$,

$$\begin{aligned} x'&= bx\min \left( 1-\frac{x}{K}, 1-\frac{x}{(P-\theta y)/q}\right) - f(x) y \\&\le bx\min \left( 1-\frac{x}{K}, 1-\frac{x}{(P-\theta y)/q}\right) \\&\le bx\min \left( 1-\frac{x}{K}, 1-\frac{x}{P/q}\right) \\&\le bx\min \left( 1-\frac{x}{k}\right) \end{aligned}$$

The differential equation simplifies to:

$$x(t)\le \frac{kx(0)}{(k-x(0))e^{-bt}+x(0)}$$

This implies that $x(t_1)\le \frac{kx(0)}{(k-x(0))e^{-bt_1}+x(0)}<k$, contradicting the assumption that $x(t_1)=k$. Thus, no trajectory can touch the right-hand-side boundary of the rectangle.

Case 3: $y(t_1) = 0$. We assume that the trajectory reaches the bottom boundary of the rectangle. Then for every $t\in [0,t_1]$,

$$\begin{aligned} y'&= \hat{e}\min \left( 1,\frac{(P-\theta y)/x}{\theta }\right) f(x) y - \xi y - d y \ge -dy \end{aligned}$$

This implies that $y(t_1)\ge y(0)e^{-dt_1}>0$, contradicting the assumption that $y(t_1)=0$. Thus, no trajectory can touch the bottom boundary of the rectangle.

Case 4: $y(t_1) = \frac{P}{\theta }$. We assume that the trajectory reaches the top boundary of the rectangle. Then for every $t\in [0,t_1]$,

$$\begin{aligned} y'&= \hat{e}\min \left( 1,\frac{(P-\theta y)/x}{\theta }\right) f(x) y - \xi y - d y \\&\le \hat{e} \left( \frac{(P-\theta y)/x}{\theta }\right) f(x) y \\&\le \hat{e} f'(0) y\left( \frac{P}{\theta }-y\right) = \hat{e} f'(0)\frac{P}{\theta }y\left( 1-\frac{y}{P/\theta }\right) \end{aligned}$$

Similar to case 2, we find that $y(t_1)<\frac{P}{\theta }$, contradicting the assumption that $y(t_1)=\frac{P}{\theta }$. Thus, no trajectory can touch the top boundary of the rectangle. $\square$

### Phase Plane for Base Model

Phase plane analysis for base model (1) for varying *K* values are presented in Fig. 5. Here, when $K=0.25$, numerical trajectories show that an interior equilibrium is stable. When $K=1$, there is an unstable interior equilibrium, and trajectories approach a stable limit cycle. When $K=1.5$, there are two unstable interior equilibria and one stable one, solutions approach the stable equilibrium.

## Adaptive Model Analysis

### Proof of Theorem 4

#### Proof

Let $(x(t),y(t), \xi (t))$ be a solution of system (4) with initial conditions $(x(0),y(0), \xi (0) \in \Delta$. Assume, for contradiction, that there exists a time $t_1>0$, such that the trajectory with initial conditions in the open region touches or crosses the boundary of the closed region for the first time. Similar arguments to those made in the proof of Theorem 1 (Appendix A.1) can be made to ensure that *x* remains in (0, *k*) and *y* remains in $(0,\frac{P}{\theta })$. Two remaining cases are shown below:

Case: $\xi (t_1) = \xi _{min}$. We assume that the trajectory reaches the lower boundary of $\xi$. Then for every $t\in [0,t_1]$,

$$ \frac{d\xi }{dt}= (\xi _{max}-\xi )(\xi - \xi _{min})v\left[ \hat{e}\min \left( 1,\frac{(P-\theta y)/x}{\theta }\right) \frac{\mu x}{(1+\mu \xi \tau x)^2} - 1 \right] =0 $$

and the trajectory can not cross this boundary.

Case: $\xi (t_1) = \xi _{max}$. Similar to the case above, the trajectory can not cross this boundary. Thus, any solution starting in $\Delta$ will remain there for all forward time. $\square$

### Proof of Theorem 6

#### Proof

At $E_1$, the Jacobian takes the form

$$ \mathbf {J|_{E_1}} = \begin{bmatrix} -b & kF_y(k,0,\xi ^*) & kF_{\xi }(k,0,\xi ^*) \\ 0 & G(k,0,\xi ^*) & 0 \\ vH_x(k,0,\xi ^*) & vH_y(k,0,\xi ^*) & vH_{\xi }(k,0,\xi ^*) \end{bmatrix} $$

where

$$\begin{aligned} k&=\min \left( K,\frac{P}{q}\right) \\ F_y(k,0,\xi ^*)&= {\left\{ \begin{array}{ll} \displaystyle \frac{-\mu \xi ^*}{1+\mu \xi ^*\tau k}< 0,& \text {if } K<\frac{P}{q}\\ \\ \displaystyle -\frac{bkq\theta }{P^2}\frac{-\mu \xi ^*}{1+\mu \xi ^*\tau k}<0, & \text {if } K>\frac{P}{q} \end{array}\right. }\\ \\ F_\xi (k,0,\xi ^*)&= 0\\ \\ G(k,0,\xi ^*)&= \hat{e}\min \left( 1,\frac{P}{k\theta }\right) \left( \frac{\mu \xi ^* k}{1+\mu \xi ^*\tau k}\right) - \xi ^* - d\\ \\ H_x(k,0,\xi ^*)&= {\left\{ \begin{array}{ll} \displaystyle (\xi _{max}-\xi ^*)(\xi ^*-\xi _{min})\left[ -\frac{2\hat{e}\mu ^2\xi ^{*2}\tau }{(1+\mu \xi ^*\tau k)^3}\right]< 0,& \text {if } 1<\frac{P}{k\theta }\\ \\ \displaystyle (\xi _{max}-\xi ^*)(\xi ^*-\xi _{min})\left( \frac{P\hat{e}}{k\theta }\right) \left( \frac{\mu \xi ^{*}}{(1+\mu \xi ^*\tau k)^2}\right) \left[ -\frac{1}{k}-\frac{2\mu \xi ^*\tau }{1+\mu \xi ^*\tau k}\right]< 0,& \text {if } 1>\frac{P}{k\theta } \end{array}\right. }\\ \\ H_y(k,0,\xi ^*)&= {\left\{ \begin{array}{ll} \displaystyle 0,& \text {if } 1<\frac{P}{k\theta }\\ \\ \displaystyle (\xi _{max}-\xi ^*)(\xi ^*-\xi _{min})\left( -\frac{\hat{e}}{k}\right) \left( \frac{\mu \xi ^{*}}{(1+\mu \xi ^* \tau k)^2}\right) < 0,& \text {if } 1>\frac{P}{k\theta } \end{array}\right. } \end{aligned}$$

$$\begin{aligned} H_{\xi }(k,0,\xi ^*) =&-(\xi ^*-\xi _{min})\left[ \frac{\hat{e}\min \left( 1,\frac{P}{k\theta }\right) \mu \xi ^*}{\left( 1+\mu \xi ^*\tau k\right) ^2}-1\right] + (\xi _{max}-\xi ^*)\left[ \frac{\hat{e}\min \left( 1,\frac{P}{k\theta }\right) \mu \xi ^*}{\left( 1+\mu \xi ^*\tau k\right) ^2}-1\right] \\&+ (\xi _{max}-\xi ^*)(\xi ^*-\xi _{min})\left[ \hat{e}\min \left( 1,\frac{P}{k\theta }\right) \right] \left[ \frac{\mu -\mu ^2\xi ^*\tau k}{(1+\mu \xi ^*\tau k)^3}\right] \end{aligned}$$

$$ \mathbf {J|_{E_1}} = \begin{bmatrix} -b & kF_y(k,0,\xi ^*) & 0 \\ 0 & G(k,0,\xi ^*) & 0 \\ vH_x(k,0,\xi ^*) & vH_y(k,0,\xi ^*) & vH_{\xi }(k,0,\xi ^*) \end{bmatrix} $$

Therefore, eigenvalues must satisfy the following

$$(\lambda +b)(\lambda -G(k,0,\xi ^*))(\lambda -vH_{\xi }(k,0,\xi ^*))=0 $$

and take values on the diagonal, $\lambda =-b, G(k,0,\xi ^*)$, and $vH_{\xi }(k,0,\xi ^*)$. If $G(k,0,\xi ^*)<0$ and $H_{\xi }(k,0,\xi ^*)<0$, then the eigenvalues of $\mathbf {J|_{E_1}}$ are negative, yielding that $E_1$ is stable. $\square$

### Plot of $H_{\xi }(k,0,\xi ^*)$ for Theorem 6

Regions where $H_{\xi }(k, 0, \xi ^*) < 0$ in $(K, \xi ^*)$ parameter space is shown in Fig. 6a. The equilibrium $E_1=(k,0,\xi ^*)$ lies on the black curves, and here $\xi ^*$ corresponds to the values of $\xi$ where $\frac{d\xi }{dt} = 0$, which could be the maximum feeding effort ($\xi _{\max }$), minimum feeding effort ($\xi _{\min }$), or intermediate values of $\xi$ when *K* is low. The light gray region in Fig. 6a represents areas where $H_{\xi }(k, 0, \xi ^*) < 0$, indicating possible stable conditions for the grazer extinction equilibrium $E_1$ provided that $G(k, 0, \xi ^*) < 0$ is also satisfied.

In Fig. 6b, we plotted the regions where $H_{\xi }(k, 0, \xi ^*) < 0$ and $G(k, 0, \xi ^*) < 0$ in the $(K, \xi ^*)$ parameter space and highlighted where both condition hold (dark gray regions). The dark gray regions, which represent areas where both conditions hold, indicate stable conditions for the grazer extinction equilibrium $E_1$. For high values of *K*, $\xi ^*=\xi _{\max }$ or $\xi _{\min }$, however when $\xi ^*=\xi _{\max }$, $E_1=(k, 0, \xi _{\max })$ is unstable and when $\xi ^*=\xi _{\min }$, $E_1=(k, 0, \xi _{\min })$ is stable. For lower values of *K* there are some intermediate values of $\xi ^*$. Here, there are values where $E_1=(k, 0, \xi ^*)$ can be unstable, where the grazer eats enough, but not too much, and balances the cost of feeding efforts.

### Interior Eqilibria Analysis of Adaptive Model

Phase portrait analysis for the adaptive model (1) for varying *K* values are presented in Fig. 7. Here, when $K=0.25$, numerical trajectories show that an interior equilibrium is stable. When $K=1$, trajectories approach a stable limit cycle. When $K=1.5$ solutions trajectories approach another stable interior equilibrium.
